# Perioperative POCUS: Technical Considerations and Applications for the Pediatric Anesthesiologist

**DOI:** 10.3390/jcm15176539

**Published:** 2026-08-24

**Authors:** Allison Heizelman, Rebecca Evans, Emmeline Chuu, Ying Eva Lu-Boettcher

**Affiliations:** 1 Department of Anesthesiology, University of Wisconsin School of Medicine and Public Health, Madison, WI 53726, USAluboettcher@wisc.edu (Y.E.L.-B.); 2 Anesthesia Care Associates Medical Group, San Francisco, CA 94118, USA

**Keywords:** point-of-care ultrasound, pediatric anesthesiology, cardiac ultrasound, lung ultrasound, FAST, airway ultrasound, gastric ultrasound, perioperative anesthesia

## Abstract

**Background**: Point-of-care ultrasound (POCUS) use continues to expand across specialties caring for pediatric patients in the perioperative period, offering noninvasive, diagnostic information that complements clinical assessment in real time. Although many POCUS applications were developed and validated in adults, pediatric POCUS requires unique considerations. This review examines the current evidence of diagnostic POCUS within pediatric perioperative care. **Methods**: A structured search of PubMed was performed for literature published through December 2025, using terms related to point-of-care ultrasound, perioperative care, cardiac, lung, gastric, and airway ultrasound, as well as focused assessment with sonography in trauma (FAST). Priority was given to systematic reviews, meta-analyses, randomized controlled trials, clinical practice guidelines, and landmark observational studies, while supplemented by recent high-quality publications. **Findings**: POCUS in each of the following areas is discussed: cardiac, pulmonary, airway, gastric, and FAST. Topics for each of the areas include a general overview, considerations for devices and probe selection to optimize imaging, specific uses and applications of the imaging, and potential limitations. **Conclusions**: POCUS is a valuable tool to complement comprehensive clinical evaluation of children in the perioperative period. Widespread adoption will require additional pediatric normative data, standardized protocols, and structured training for providers. Future research should continue to validate POCUS algorithms and clarify the role of artificial intelligence in enhancing image interpretation and accessibility.

## 1. Introduction

Point-of-care ultrasound (POCUS) use continues to expand across specialties caring for pediatric patients by providing noninvasive, real-time diagnostic information that can complement clinical assessment and standard monitoring. Although many POCUS applications were initially developed and validated in adults, pediatric POCUS requires unique considerations and cannot be directly extrapolated from adult practice. Differences in body size, transducer selection, developmental anatomy, normal physiologic parameters, heart and respiratory rates, disease prevalence, patient cooperation, and the availability of pediatric-specific validation studies significantly influence image acquisition, interpretation, and clinical application.

This review aims to examine the current evidence, limitations, and practical integration of diagnostic POCUS within pediatric perioperative care. The discussion focuses on the evolving role of POCUS in pediatric anesthesiology, including cardiac, pulmonary, airway, gastric, and focused assessment with sonography in trauma (FAST) applications. Ultrasound use for regional anesthesia and vascular access is beyond the scope of this review. By highlighting both established applications and areas requiring further investigation, this review seeks to provide a framework for the safe and effective incorporation of POCUS into pediatric perioperative practice.

## 2. Methods

To ensure a comprehensive and transparent review of the literature, we performed a structured search of PubMed through the Ebling Library. The search included studies published from database inception through December 2025 and focused on the use of POCUS in the perioperative setting. Search terms combined controlled vocabulary and keywords related to “point-of-care ultrasound,” “perioperative ultrasound,” “pediatric anesthesia,” “cardiac ultrasound,” “lung ultrasound,” “gastric ultrasound,” “airway ultrasound,” and “focused assessment with sonography in trauma.” Reference lists of relevant articles and recent reviews were also screened to identify additional pertinent studies. Priority was given to systematic reviews, meta-analyses, randomized controlled trials, clinical practice guidelines, and landmark observational studies, while incorporating recent high-quality publications to ensure that the review reflects the current state of the evidence. This search strategy was intended to provide a balanced and comprehensive synthesis of contemporary literature.

## 3. Cardiac Evaluation

Focused cardiac point-of-care ultrasound (C-POCUS) allows clinicians to evaluate the heart using a defined and limited number of transthoracic echocardiographic views [1]. It is currently utilized by providers across multiple pediatric subspecialties, including critical care, emergency medicine, cardiology and anesthesiology. Optimal use of C-POCUS involves a targeted clinical question and integration with other clinical data, such as physical examination findings and vital signs, particularly in patients with presumed normal cardiac anatomy [2]. There have been detailed guidelines for C-POCUS in children published in 2023 by the American Society of Echocardiography which encompass indications, equipment and technical considerations, overview of imaging recommendations and training with competency assessment [2].

Cardiac POCUS has helped guide the management of acute hemodynamic instability and shock and provide additional assessment during circulatory arrest [1]. C-POCUS should not be confused with echocardiography or considered a replacement for formal echocardiography, which encompasses a more complete set of images and evaluations [2,3,4]. C-POCUS is also not recommended for the diagnosis of congenital heart disease, which requires detailed cardiology evaluation, although it may assist in identifying patients who warrant further investigation [2,4]. Of note, while pre-operative evaluation of the heart has been described in adult patients, the role of C-POCUS in non-emergent situations for pediatric patients remains to be defined [2].

Ultrasound machines used for C-POCUS in children should ideally have both low- and high-frequency probes due to the wide range of patient sizes and depths encountered in the pediatric population [2,5]. While there is variation in the frequencies recommended, lower frequencies of ~2–4 MHz are more ideal for larger and adolescent patients. Higher frequencies between 4.5 to ≥7.5 MHz are recommended for smaller patients, including infants, toddlers, and children up to 12 years of age [2,5]. If lower frequency probes are used for smaller patients, there are potential pitfalls. The larger probe footprint can prevent adequately scanning between narrow rib spaces. Also, shallow, near-field imaging can have poorer image quality, missing pathology and affecting imaging interpretation [2]. If only lower frequency transducers are available, focusing on the subcostal view is recommended, using the liver as an acoustic window with the heart visualized in a farther field to enhance imaging quality [2]. Recommended views are similar for those in the adult population: subcostal view, subcostal inferior vena cava (IVC) view, apical four-chamber view, parasternal long-axis view and parasternal short-axis view. Covering each of these views individually is outside the scope of this publication.

C-POCUS is highly useful in the rapid assessment of acute hemodynamic instability and shock states [1,2,3]. It has been shown to reduce the time to make a definitive diagnosis and initiate appropriate treatments in these critical scenarios [5]. Clinicians can rapidly and non-invasively assess a variety of factors such as overall cardiac function, left ventricular and right ventricular size, assessment of intraventricular volume status, presence of pericardial effusion and gross valvular abnormalities [2,5,6]. Undifferentiated hypotension and tachycardia unresponsive to routine interventions are some of the most common indications for C-POCUS in pediatrics [5]. Volume status and fluid responsiveness is commonly assessed in these critical situations by indirect measures including physiologic parameters (such as blood pressure, heart rate, or capillary refill time) and invasive monitoring (such as central venous catheterization and central venous pressure monitoring) [2,3]. In pediatric patients, C-POCUS can reveal findings such as ventricular cavity obliteration (“kissing ventricles”), suggestive of hypovolemia, or right ventricular dilation, indicating volume overload. A key distinction between adults and children is that hypotension of primary myocardial origin in pediatric patients typically presents as global cardiac dysfunction rather than focal regional wall motion abnormalities [5]. C-POCUS has also been described incorrectly, confirming peripheral intravenous access location by the detection of micro-bubble turbulence in the right heart following an injection of saline through the catheter [7]. Some important C-POCUS uses and clinical findings are summarized in Table 1.

C-POCUS has been utilized during and after cardiac arrest; however, it is not currently included in pediatric-specific resuscitation algorithms [8]. It can potentially identify reversible causes of cardiac arrest (such as pericardial tamponade, hypovolemia, or pulmonary embolus) or presence of intrinsic cardiac activity [3,4,8,9,10]. However insufficient evidence currently exists to recommend for or against routine use of C-POCUS during pediatric cardiac arrest, weighing its use against known deleterious consequences of interrupting chest compressions [1,4,11]. The view most commonly utilized in the adult population is the subcostal four-chamber view obtained during a pulse check, as this is the least disruptive for chest compressions [10]. Palpation of pulses in unconscious children can be unreliable even by experienced healthcare providers, so C-POCUS has been described to assist in demonstrating presence or absence of a cardiac pulse. However, further research is required to fully establish its efficacy and impact on survival [5,12].

It is important to recognize limitations of C-POCUS in children. There is significant variation in ultrasound devices and the quality of imaging produced affecting imaging interpretation [2]. Children, especially infants, have faster heart rates, thus requiring higher frame rates for optimal image resolution. In images with more shallow depth, frame rate is automatically increased; therefore, adjusting the window to the minimal acceptable depth is important to optimize image quality [5]. Pediatric patients may also have limited windows due to narrow spacing between ribs, difficulty with positioning, underlying clinical conditions (i.e., rib cage deformities, contractures) and limited patient cooperation with exams [2,5]. Children may also not be amenable to maneuvers that can improve imaging, such as deep inspiration or Valsalva maneuver, which can displace the heart downward, improving the subcostal four-chamber view [5]. When imaging is attempted intraoperatively, limited access to the patient and inability to change positioning can create challenges with image acquisition and limit the quality of images [5].

As with any imaging modality, there is concern for exam accuracy to avoid diagnostic error; however, there is growing evidence that C-POCUS can be performed in children appropriately by non-cardiologists in various settings [1]. Literature supports that C-POCUS can be performed accurately even for novice trainees, especially when dichotomous or semiquantitative methods of assessment are used (e.g., present or absent, normal or depressed) [2]. Spurney et al. found that 90% of scans performed in the pediatric intensive care unit and emergency department settings evaluating ventricular size and function and presence of pericardial effusion had similar findings between the novice and experts [13]. It has also been shown that physicians in the pediatric emergency department can accurately assess volume status, global systolic dysfunction and pericardial effusion with proper training [14,15]. Miller et al. demonstrated real-time C-POCUS interpretation had a sensitivity and specificity of 100% and 99.5%, respectively, for both pericardial effusion and left ventricular systolic dysfunction when trained pediatric emergency medicine attendings were compared with expert POCUS reviewers [14]. In addition, creation and implementation of C-POCUS protocols in the pediatrics emergency department can yield accurate imaging interpretation and exam results [16]. While there is published literature from other pediatric specialties on the accuracy, utility and efficacy of C-POCUS imaging, further studies need to be done specifically in pediatric anesthesia and the perioperative period.

## 4. Pulmonary Evaluation

Lung POCUS aids the real-time diagnosis of acute conditions without the delay and radiation exposure often associated with traditional pulmonary imaging modalities. Diagnostic applications in anesthesiology are broad, though useful for confirmation of lung isolation and diagnosing decreases in oxygen saturation accompanied by deteriorating vital signs [17,18].

Equipment selection can significantly impact the diagnostic accuracy of pediatric lung POCUS. Infants and children often have thinner chest walls compared to adults, requiring a probe that has adequate image resolution at a shallower depth; therefore, a higher-frequency, linear probe is recommended to allow for better resolution of superficial structures, including the pleural line [19]. For larger children and adolescents, or when deeper penetration is required, a lower frequency curvilinear with frequencies around 3–5 MHz can be used [19].

There are important age-related considerations that may affect lung ultrasound interpretation, most notably in the neonatal and infant populations. Due to a remarkably compliant chest wall, chest wall movement can create a false impression of pleural lung sliding, even when true pleural sliding is absent. This could lead to false-negative findings for pneumothorax or difficulty when confirming lung isolation in one-lung ventilation.

Pediatric anesthesiologists can utilize lung POCUS for assessment of lung isolation and identifying acute pathologies. During procedures that require one-lung ventilation, traditional methods have used auscultation and flexible bronchoscopy to confirm lung isolation [17]. A recent prospectively blinded feasibility study including 40 patients demonstrated that lung ultrasound had a diagnostic accuracy of 95% for confirming effective single-lung ventilation in pediatric thoracic surgery, significantly outperforming auscultation, which only had 68% accuracy [17]. While appearing promising, this represents preliminary evidence and there are limited additional publications evaluating one-lung ventilation with POCUS in infants and children [20]. Therefore, further multicenter validation would be needed before widespread enactment can be recommended.

Despite its utility, lung POCUS in pediatric patients has several important limitations. As with other POCUS scans, technique is operator-dependent, requiring appropriate training and experience for both image acquisition and interpretation [3]. In addition, image quality and diagnostic accuracy can vary significantly between different ultrasound machines and transducers, with low-end equipment potentially failing to detect subtle findings, as demonstrated by diagnostic difference between respiratory distress syndrome and transient tachypnea of the newborn [21]. Further, lung POCUS can only visualize pathology that extends to the pleural surface, missing central consolidations or masses that do not reach the pleura; however, studies suggest that over 95% of pathological changes have a pleural component in pediatric patients [22]. And with other imaging and procedures performed in the pediatric population, technical challenges in image acquisition related to uncooperative or agitated children and positioning challenges can arise [3,18].

Outside of the perioperative setting, there is widespread use of lung ultrasonography in other specialties. Pediatric emergency medicine physicians routinely use lung ultrasound to diagnose pneumonia with improved sensitivity and similar specificity compared to chest radiograph while reducing radiation exposure in pediatric patients [22]. Neonatologists have incorporated lung ultrasound into assessments for pulmonary surfactant deficiency assessment, respiratory distress syndrome, pneumothoraces, and ventilator-associated lung injury [23]. To standardize lung POCUS techniques and interpretation, the European Society of Pediatric and Neonatal Intensive Care (ESPNIC) published comprehensive evidence-based recommendations in 2020 after a rigorous review with international recommendations [24]. Continued development of consensus reviews substantially improves consistency and reliability across specialties, facilitating broader implementation in clinical practice, particularly in pediatric anesthesia. As evidence continues to accumulate supporting its clinical utility, lung POCUS is likely to become increasingly integrated into standard anesthesiology practice for pediatric patients.

## 5. Airway Evaluation

POCUS has gained popularity as a tool to facilitate safer airway management in recent years. Pediatric POCUS has been used as an adjunct to (1). optimize endotracheal tube (ETT) insertion depth, (2). predict difficult laryngoscopy, (3). aid in ETT size selection, and (4). aid in confirmation of endotracheal intubation, particularly in settings where end-tidal CO_2_ is not reliably detected [1,25,26,27,28]. Moreover, integrating airway and lung POCUS during unexpected difficulty with ventilation enables rapid differentiation among esophageal intubation, mainstem bronchial intubation, and pneumothorax, highlighting the value of a multi-organ ultrasound approach for perioperative crisis management. An overview of clinical applications, ultrasound findings and benefits of airway ultrasound are summarized in Table 2.

Data in pediatric patients undergoing elective procedures suggest that incorrect ETT placement depth (either too shallow or too deep) can occur in up to 18% of intubations despite confirmation of bilateral breath sounds, bilateral chest rise, and adequate end-tidal CO_2_ by the anesthesia team [29]. The younger the patient, the higher the likelihood of ETT malposition. In fact, Harris et al. reported a peak incidence of ETT malposition in 35% of intubations in children less than 1 year old. The incidence decreased with increasing age but remains over 10% until the age of ten [29]. Common methods and calculations for identifying correct ETT depth range from 42% to 73% in success rates [30]. Chest radiography is impractical as the gold standard; thus, POCUS may be a useful adjunct. A study by Mori et al. found that a three-point technique (transtracheal and bilateral lung sliding visualization) resulted in a 85.7% sensitivity and 98.3% specificity for mainstem intubation in the pediatric emergency department setting [25]. In pediatric patients undergoing elective general anesthesia, a study by Uya et al. demonstrated that ultrasound of saline-filled ETT cuff at the suprasternal notch corresponded with a 95% accuracy in correct endotracheal tube depth on chest radiographs [31]. In another pediatric anesthesia study, Ramsingh et al. reported a robust correlation between POCUS and chest radiographic measurements of the distance between ETT cuff to the cricoid cartilage, further demonstrating the potential value of ultrasound assessment of ETT depth in anesthesia [32].

POCUS has also shown promise as an adjunct for pediatric difficult laryngoscopy assessment using hyomental distance (HMD) [33]. In a study by Zheng et al. that examined US measurements of HMD in the extended position and its ability to predict difficult laryngoscopy, the authors reported a sensitivity of 88% and specificity of 60% for 5–8 year olds, and a sensitivity of 71% and specificity of 83% for 9–12 year olds [34]. In another study in children undergoing anesthesia under two years old, Kahlon et al. examined US evaluation of HMD ratio between neutral position and extended position and its ability to predict difficult laryngoscopy. The authors reported that HMD ratio significantly varied between children with easy and difficult laryngoscopy in this patient population [35]. Of note, further validation and establishment of standardized protocols are needed to determine the optimal ultrasound predictors for difficult intubation in the pediatric population [33]. Current evidence is largely derived from small, single-center studies with heterogeneity in patient populations, age groups, ultrasound techniques, anatomical measurements, positioning, and definitions of difficult laryngoscopy.

Ultrasonography has been used to aid in ETT size selection by measuring a child’s subglottic transverse diameter and comparing this to ETT outer diameters for the most appropriate sizing [33]. Several studies of pediatric patients undergoing general anesthesia have demonstrated that ultrasound measurement methods yielded better accuracy in determining appropriate ETT size compared to age-based ETT sizing formulas [36,37,38]. In these studies, ultrasound methods identified the correct-sized ETT between 88% and 98% of the time for cuffed ETTs (70% to 96% for uncuffed ETTs) [36,37,38].

**Table 2:** Airway POCUS Clinical Application Summary [30,31,32,33,34,35,36,37,38,39].

Clinical Application	Ultrasound Assessment	Clinical Benefit
ETT depth confirmation	Visualization of ETT cuff at the suprasternal notch; assessment of bilateral lung sliding and transtracheal views	Identifies shallow or deep ETT placement and may reduce malposition
Confirmation of tracheal vs. esophageal intubation	Transverse neck ultrasound showing ETT within the trachea; detection of “double trachea” sign	Provides rapid confirmation of ETT location, especially when end-tidal CO_2_ is unreliable (e.g., cardiac arrest)
Assessment of difficult airway risk	Ultrasound measurements of hyomental distance and hyomental distance ratio	May assist prediction of difficult laryngoscopy; further pediatric validation is needed
ETT size selection	Measurement of subglottic transverse diameter compared with ETT outer diameter	Improves prediction of appropriate ETT size compared with age-based formulas

Lastly, accidental esophageal intubation can occur in up to 21% of infants with a 3% incidence of chest compressions [18]. POCUS can be utilized to aid in confirmation of ETT location in conjunction with waveform capnography, auscultation of bilateral breath sounds, and misting of the ETT. In addition, POCUS can be helpful when expired CO_2_ levels are low and capnography can yield false negatives, such as during cardiac arrest [26]. Imaging a pediatric patient’s neck transversely above the sternal notch can yield both images of the trachea and esophagus. Visualizing two circular structures called the “double trachea sign” post-intubation would indicate esophageal intubation [39]. Pediatric data demonstrate sensitivity and specificity of 98.5–100% and 75–100%, for diagnosis of esophageal intubation, respectively [18].

## 6. Gastric Evaluation

Pulmonary aspiration of gastric contents remains a concern for pediatric anesthesiologists striving to minimize the morbidity and mortality of the pediatric perioperative patient. The incidence of pulmonary aspiration in the perioperative pediatric patient is observed to be between 2 and 10.2 per 10,000 cases, with cases often resulting in an escalation of care and/or patient harm [40,41,42,43]. Gastric ultrasound is a tool to augment the history and physical exam to facilitate clinical decision making regarding the timing and approach to providing anesthetic care.

Multiple descriptions and guides on how to perform gastric ultrasound have been previously described [44,45] with the use of gastric ultrasound in the perioperative setting specifically being first described in 2009 in the groundbreaking article, “Ultrasound assessment of gastric content and volume” [44]. This article included only adult age patients; however, newer data is now available for pediatric specific evaluation and parameters. Qualitative assessment of gastric content is described with a simple grading system ranging from grade 0 (no contents in the antrum) to grade 2 (gastric fluid observed in the antrum in the supine and right lateral decubitus positions) [46]. This qualitative scale was enhanced in additional articles to include the antral shape, thickness of the antral wall, presence or absence of peristalsis, and the quality of the content of the stomach (hyper/hypoechoic/heterogeneous) [44,47].

**Figure 1:**
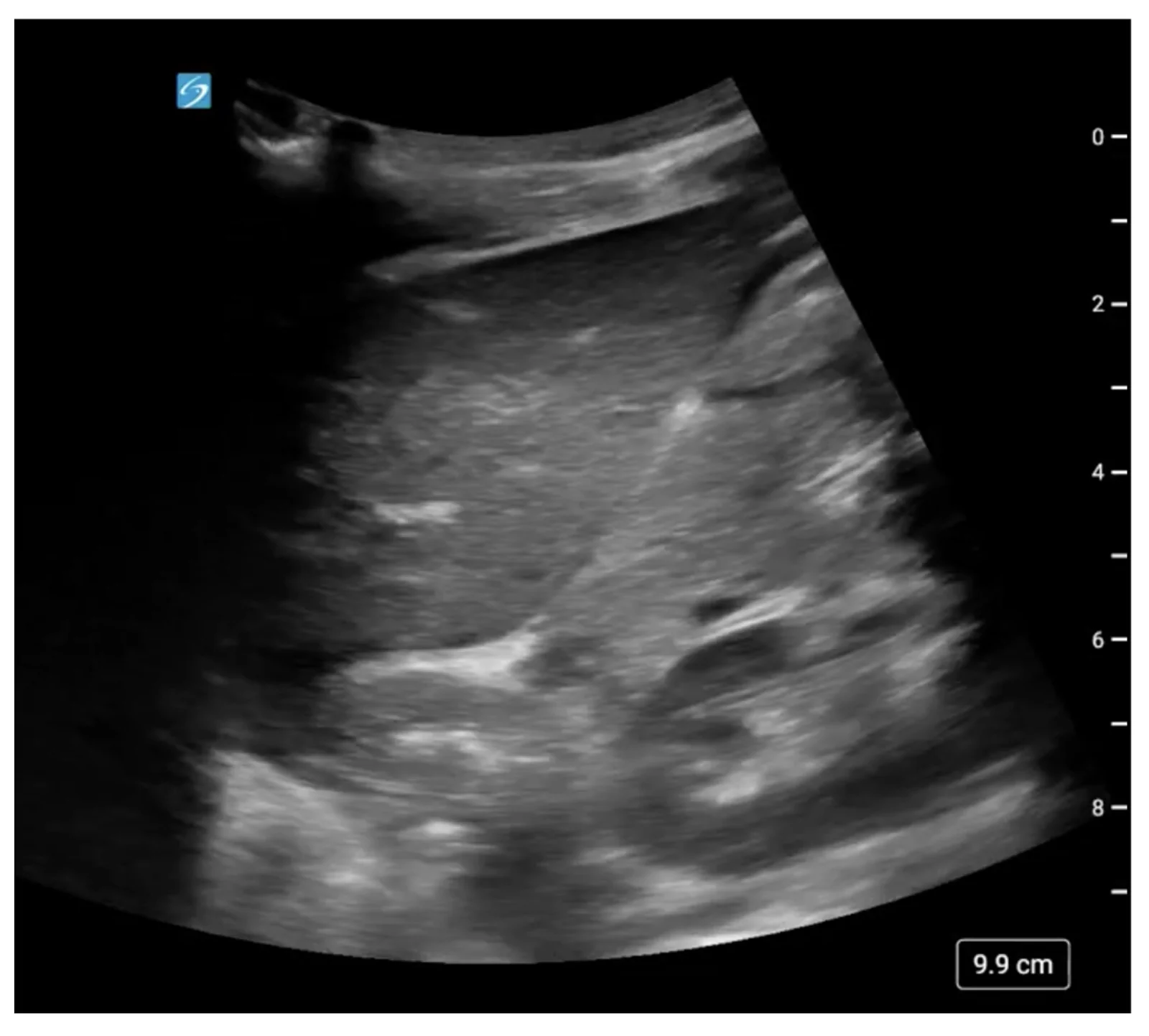
Use of curvilinear probe to obtain gastric imaging.

**Figure 2:**
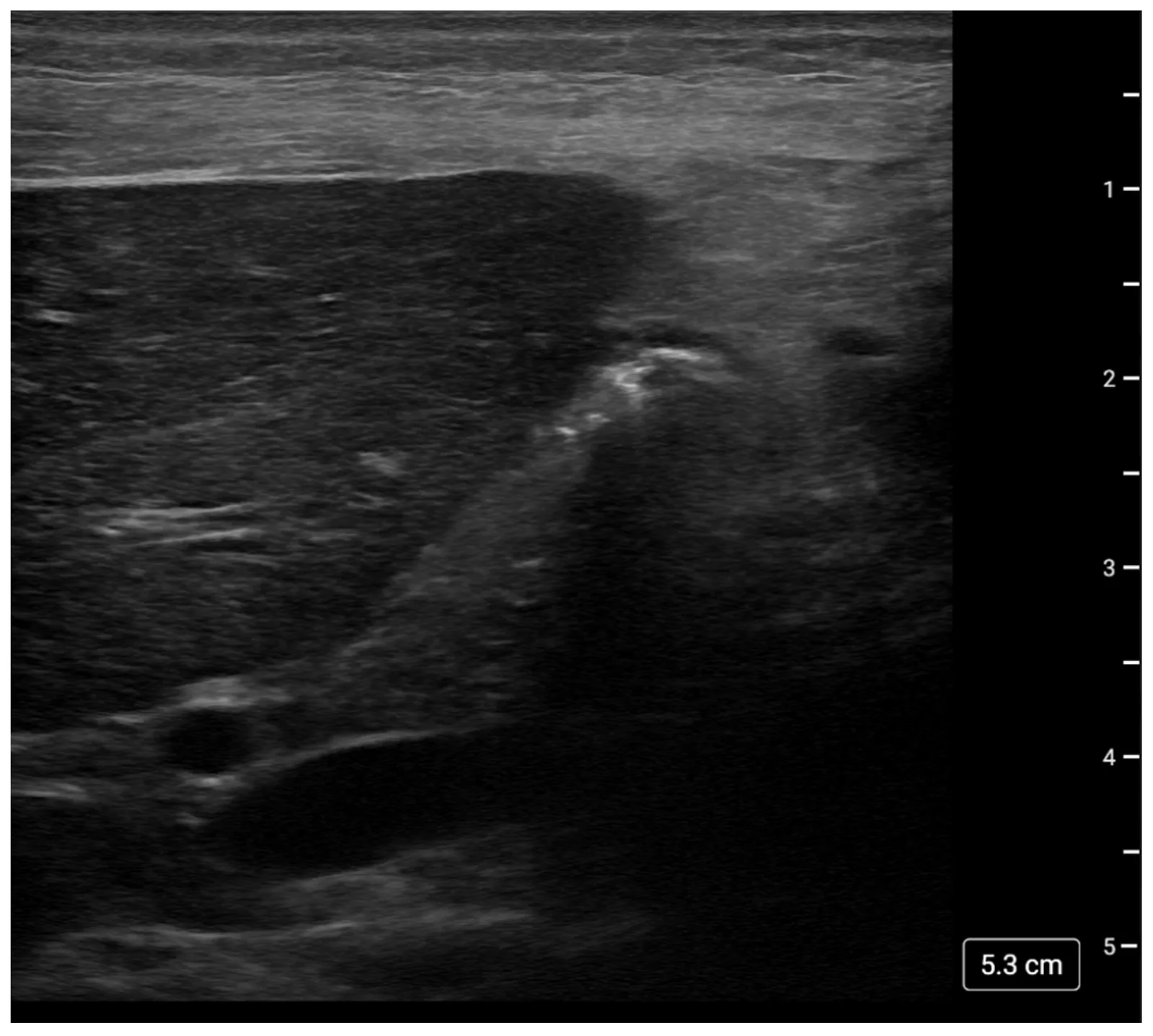
Use of linear probe to obtain gastric imaging.

One caveat to performing gastric ultrasound on pediatric patients involves the choice of transducer to obtain optimal imaging of deep structures such as the abdominal aorta. Pediatric patients of elementary school age or smaller benefit from the higher resolution of a linear 5–13 Hz probe; young adults or older are best imaged with a curvilinear 2–5 Hz probe [45]. Patient position and views obtained remain the same regardless of patient age or size. Gastric imaging of a 7-year-old, 19 kg child was obtained with both curvilinear (5–1 MHz, Sonosite) and linear (15–4 MHz, Sonosite) probes to show the differing appearance related to the device used (Figure 1 and Figure 2, respectively).

Mathematical modeling has allowed us to quantify the volume of gastric contents, with initial models being developed and refined for adult patients [44,47,48]; however, further refinement for the pediatric patient now exists. A publication by Spencer et al. from 2015 determined the baseline amount of gastric secretions of a fasted pediatric patient undergoing endoscopy to be ≤1.25 mL/kg, with volumes > 1.25 mL/kg considered a full stomach. A formula was then created to model the calculated gastric volume in a pediatric patient:

Gastric volume (mL) = −7.8 + (3.5 × RLD CSA) + (0.127 × age),

with RLD CSA being the right lateral decubitus cross sectional area of the antrum (cm^2^) and age being in months [49].

It is important to note this model was created from a specific population (i.e., pediatric patients undergoing endoscopy) and is not scientifically validated for application to a broader pediatric population. Therefore, this formula may not be sufficient for assessing all pediatric patients presenting for anesthetic procedures, including those with a high risk for aspiration such as urgent/emergent procedures, delayed gastric emptying due to medications and/or pathophysiology, feeding intolerance, enteral nutrition, obesity, and/or medical complexity (ASA III or higher) [40,42,43,50]. This model also should not be used as an absolute definition of “full stomach”, as there is no certainty between measured gastric content and aspiration risk.

Gastric ultrasound has been used in the setting of sedation by emergency room providers outside of the perioperative space. In contrast to the fasting guidelines established by the American Society of Anesthesiologist prior to a procedure, the American College of Emergency Physicians has the following recommendation: “Do not delay procedural sedation in adults or pediatrics in the ED based on fasting time” [51]. Numerous studies have identified pediatric patients as having a full stomach and a “high risk” potential for aspiration using gastric ultrasound prior to sedation, with no adverse events reported [52,53,54,55,56]. Possible explanations for these observations may include the health status of the child prior to injury, maintenance of airway reflexes under sedation, and/or duration of sedation.

Gastric POCUS use crosses many disciplines with applications outside of the operating room that may be transferable to the perioperative space. Procedurally, gastric and esophageal ultrasound have been used successfully to facilitate placement of oro- and nasogastric tubes [57]. Gastric ultrasound has also been used to confirm correct placement of orogastric tubes, nasogastric tubes, G-tubes, and GJ-tubes, reducing the patient’s exposure to radiation and the cost of the procedure [58,59,60,61]. Gastric ultrasound can also be used as a diagnostic tool. For example, emergency room physicians are using gastric ultrasound for foreign body assessment, allowing for the identification of objects that are not radiopaque and serial assessment of the objects without radiation exposure to the patient [62]. It has also been used in evaluating the impact of mask ventilation versus mechanical ventilation on gastric insufflation for patients in the pediatric intensive care unit. Insufflation was noted with mask ventilation pressures as low as 14 cmH_2_O and was also found in some mechanically ventilated patients, though to a significantly lesser degree than those undergoing mask ventilation [62].

## 7. Abdominal Evaluation in Trauma

The Focused Assessment with Sonography in Trauma (FAST) exam helps assess trauma patients for thoracic and abdominal injuries that can cause bleeding. While its utility in adult patients has been established, its use is more variable in pediatric trauma likely due to high false-negative rates [63]. FAST traditionally evaluates for the presence of free fluid in four views: right upper quadrant (RUQ), left upper quadrant (LUQ), pelvic and cardiac [63]. The Extended Focused Assessment with Sonography in Trauma (eFAST) exam, which has now become standard practice for emergency medicine providers and in trauma centers, also includes lung ultrasound as part of the thoracic assessment [64,65]. While FAST generally has been performed in emergency room settings, findings can be relevant for pediatric anesthesiologists caring for children in the perioperative period, as the presence of intraabdominal or pelvic free fluid, pleural effusions or pericardial effusions would likely change anesthetic management.

The FAST exam has several advantages over other types of evaluations used to evaluate abdominal trauma injuries, including CT scans and diagnostic peritoneal lavage. FAST is non-invasive and spares patients ionizing radiation. It is not prohibitively time consuming, can be performed at bedside and repeated for real-time assessment changes in clinical status [65].

Probe selection for FAST exams is an important consideration in the pediatric population. To obtain FAST images in the adult population, a lower frequency probe is generally used, such as a curvilinear (2–5 MHz) or phased array (3–4.5 MHz), as this allows for the visualization of deeper structures [63,66]. Because of their smaller size, pediatric patients often require less penetration compared to adults; however, high-frequency linear probes generally do not assess free fluid at an adequate depth for a complete FAST evaluation [65].

One important note for the pediatric FAST exam should be considered: a normal exam does not exclude the presence of abdominal injury. It has been reported that approximately one-third of pediatric patients with intraabdominal injury do not develop free intraperitoneal fluid, contributing to high false-negative rates in some studies and limiting the utility of FAST imaging for detection of this type of injury [65,67,68]. While there is heterogeneity across studies, they consistently demonstrate lower sensitivity and specificity for pediatric compared to adult populations [66]. A systematic review from the Cochrane Database in 2018 reported overall lower sensitivity of pediatric FAST exams compared to exams in adult or mixed populations, reporting pooled sensitivity of 63% compared to 78%, respectively. Associated specificity was also lower in pediatric patients, 91%, compared to 97% in adult or mixed populations [69]. In some centers, this exam is being combined with other clinical evaluations, such as liver enzyme screening; however, it should not be used in isolation [65]. One additional note is that some adult literature describes sensitivity and specificity of FAST exams in categories of blunt trauma and penetrating trauma; data appears lacking in pediatrics comparing these specific categories at this time.

A study from an emergency department at a large, tertiary-care trauma center retrospectively reviewed over one-hundred positive pediatric FAST exams (RUQ, LUQ and pelvic images) and evaluated the most common locations for fluid to be located. Most commonly, fluid was detected in the pelvis (95% of scans). A total of 35% and 16.5% of scans detected fluid in the RUQ and LUQ, respectively, with the most common location outside the pelvis being at the inferior tip of the liver [70]. There is concern that trace free fluid can be considered physiologic in some pediatric patients with no specific criteria to differentiate between physiologic and pathologic presence of fluid [67].

Some studies have evaluated the FAST exam’s impact on obtaining abdominal CT imaging in children when presenting after blunt torso trauma. A multicenter, prospective observational study from the Pediatric Emergency Care Applied Research Network (PECARN) from 2014 evaluated hemodynamically stable children presenting to the emergency department for blunt torso trauma; it suggests the rate of abdominal CT imaging decreases as FAST exam use increases in those children that were considered to have a 1–10% risk of intraabdominal injury [71]. A more recent publication in 2017 performed a prospective trial randomizing hemodynamically stable children presenting after blunt trauma into either a FAST exam group or the standard of care; this study found no significant difference in the rates of abdominal CT scans or missed intraabdominal injuries, which is contrary to findings of multiple studies performed in adult populations [72]. While literature exists evaluating FAST exams in pediatric emergency medicine, further studies need to be done to evaluate the utility, efficacy, and safety of this modality in perioperative period for pediatric patients.

## 8. Conclusions

In summary, POCUS is an increasingly important tool in the care of pediatric patients, offering a noninvasive means to evaluate multiple organ systems and provide real-time information that supports perioperative clinical decision-making. Its applications in pediatric anesthesiology continue to expand, with growing evidence supporting its role in airway assessment, cardiac and pulmonary evaluation, gastric volume assessment, vascular access, and procedural guidance. Pediatric POCUS is inherently operator-dependent and should be applied with a focused clinical question in mind rather than as an independent diagnostic modality. Ultrasound findings should complement, not replace, comprehensive clinical evaluation, formal imaging studies, and consultation with relevant specialists when appropriate. As POCUS becomes more integrated into routine perioperative care, several challenges must be addressed to facilitate its widespread adoption. These include the need for validated pediatric normative data across different age groups, standardized image acquisition and interpretation protocols, and robust evidence demonstrating the impact of POCUS-guided management on clinical outcomes. Equally important is the development of structured education, competency assessment, and ongoing quality assurance programs to ensure consistent and safe implementation. International efforts, including the European Society of Paediatric and Neonatal Intensive Care (ESPNIC) recommendations and structured training initiatives such as the Children’s ACuTe Ultrasound (CACTUS) program in the United Kingdom, provide emerging frameworks for competency-based education and credentialing that may help inform future standards within pediatric anesthesiology. Continued multicenter research, collaboration among specialties, and development of standardized training and accreditation pathways will be essential to establish best practices, define the appropriate scope and limitations of POCUS, and fully realize its potential to improve perioperative care for pediatric patients.

Moreover, the role of integrated multi-organ POCUS in perioperative crisis management algorithms warrants further exploration. Combining airway, lung, cardiac, and gastric assessments may expedite diagnosis, clarify causes of physiologic instability, and guide targeted interventions. Future research should focus on validating standardized multi-organ POCUS algorithms and their impact on clinical decision-making and patient outcomes.

Lastly, artificial intelligence (AI) has the potential to transform POCUS by enhancing image acquisition, quality assessment, interpretation, and quantitative analysis. Emerging applications demonstrate the ability to assist with anatomical identification, differentiate normal from abnormal findings, and detect clinically relevant abnormalities, including cardiac dysfunction, pleural effusions, lung pathology, and congestion. AI-augmented tools may be particularly valuable in supporting novice users, reducing operator variability, and expanding access to ultrasound expertise in resource-limited settings. However, successful integration into clinical practice will require extensive rigorous independent validation, transparent and explainable algorithms, standardized guidelines, and ongoing collaboration among clinicians, researchers, industry, and policymakers [73].

## Figures and Tables

**Table 1:** Potential Uses of Cardiac POCUS [1,2,3,4,5,6,7].

Example Clinical Scenario	Potential Findings	Notes
Evaluating hypotension of unknown etiology	Depressed biventricular systolic function Ventricular enlargement Gross valvular dysfunction	Requires integration of findings with other clinical data Not a standalone diagnostic tool
Volume status evaluation	Underfilled, “kissing ventricles” suggesting hypovolemia Flat or plethoric IVC	IVC size can vary by age and patient size
Assessment for pericardial effusion and/or tamponade	Fluid present between pericardium and myocardium Indentation of the RV free wall suggestive of tamponade	Small effusions may be difficult to detect
Confirming correct location of peripheral venous access (PIV)	Presence of turbulent microbubbles in the right heart following saline injection through PIV	Imaging performed simultaneously to PIV injection May be helpful to have two operators—one performing injection while other obtaining view/assessing imaging

## Data Availability

No new data were created or analyzed in this study. Data sharing is not applicable to this article.
